# Effects of green tea on miRNA and microbiome of oral epithelium

**DOI:** 10.1038/s41598-018-22994-3

**Published:** 2018-04-12

**Authors:** Guy R. Adami, Christy C. Tangney, Jessica L. Tang, Yalu Zhou, Saba Ghaffari, Ankur Naqib, Saurabh Sinha, Stefan J. Green, Joel L. Schwartz

**Affiliations:** 1 Department of Oral Medicine & Diagnostic Sciences, Center for Molecular Biology of Oral Diseases, College of Dentistry, University of Illinois at Chicago, 801 South Paulina Street, Chicago, IL USA; 2 Department of Clinical Nutrition, College of Health Sciences, Rush University Medical Center, 1700 W Van Buren St. Suite 425, Chicago, IL USA; 3 Department of Computer Science and Carl R. Woese Institute of Genomic Biology, University of Illinois at Urbana-Champaign, 2122 Siebel Center, 201N. Goodwin Ave, Urbana, IL USA; 4 DNA Services Facility, Research Resources Center, University of Illinois at Chicago, Chicago, IL USA

## Abstract

Consumption of green tea (GT) extracts or purified catechins has shown the ability to prevent oral and other cancers and inhibit cancer progression in rodent models, but the evidence for this in humans is mixed. Working with humans, we sought to understand the source of variable responses to GT by examining its effects on oral epithelium. Lingual epithelial RNA and lingual and gingival microbiota were measured before and after 4 weeks of exposure in tobacco smokers, whom are at high risk of oral cancer. GT consumption had on average inconsistent effects on miRNA expression in the oral epithelium. Only analysis that examined paired miRNAs, showing changed and coordinated expression with GT exposure, provided evidence for a GT effect on miRNAs, identifying miRNAs co-expressed with two hubs, miR-181a-5p and 301a-3p. An examination of the microbiome on cancer prone lingual mucosa, in contrast, showed clear shifts in the relative abundance of *Streptococcus and Staphylococcus*, and other genera after GT exposure. These data support the idea that tea consumption can consistently change oral bacteria in humans, which may affect carcinogenesis, but argue that GT effects on oral epithelial miRNA expression in humans vary between individuals.

## Introduction

Green tea (GT), a beverage consumed throughout the world, contains high amounts of polyphenols, chiefly catechins, chemicals that are believed to play a role in improving health. Epidemiological research shows a possible effect for GT catechins, such as (-)-Epigallocatechin Gallate (EGCG) in the prevention of a number of cancers including lung and oral cancer, though clinical trial analysis has been confusing^1–3^. In contrast the majority of published studies in rodents provide ample evidence that GT or purified GT catechins show the ability to prevent the formation and progression of induced cancers of the oral cavity, lung, digestive tract, skin, prostate, and breast^4–9^. Various studies done in cell culture provide explanations for how EGCG or other GT components may alter carcinogenesis. These include assisting in detoxification of carcinogen oxidizing agents, changes in gene expression, induction of cell cycle arrest and apoptosis, anti-inflammatory actions, and inhibition of tumor-associated angiogenesis^10^, at times working through miRNA intermediaries^11^. Application of these results to *in vivo* experiments has been slow^12^. Plasma and cellular levels of intact catechins are often quite low due to poor uptake by gut epithelium, and the metabolized forms seen at high levels in cells *in vivo* can differ from what is studied *in vitro*^13–15^. There has been little progress in determining how GT changes global gene expression *in vivo*, information that may inform us on the mechanism of cancer inhibition. A lung cancer induction model in mice has been used to identify gene expression changes related to inflammation and regulation of cell proliferation that occur weeks after EGCG exposure in lung adenoma tissue^16^. Further analysis of miRNA expression, major regulators of gene expression in cells, in this lung model and a liver model showed changes in miRNAs with EGCG exposure^17,18^. This correlated well with the decrease in progression of adenomas to carcinoma in the lung tumor model and argues for changes in epithelial gene expression induced by GT exposure. Evidence for changes in RNA expression in humans due to GT is limited^19^.

Tobacco exposure is a major risk factor for a number of the cancers that epidemiological and rodent studies indicate GT may prevent. Tobacco can induce tumors as a mutagen, though it also causes additional changes in cellular gene expression and the way cells interact with the microenvironment, resulting in cancer^20,21^. Tobacco-induced changes in mRNA and microRNA in upper airway epithelium are well documented and include changes in xenobiotic metabolism, antioxidant- and inflammation-related genes^21–23^. Tobacco exposure is thought to prime epithelial cells for a pathway toward transformation, while there is evidence that GT can minimize *in vivo* effects of smoking, such as DNA adducts and inflammation, and increase DNA repair capability^24–28^.

Potential oral cavity effects of tea consumption associated with bacteria include reduction in inflammation, decreased periodontal disease and reduced caries^29,30^. These conditions have been linked to chronic systemic diseases such as cancer and diabetes^31^. Many studies have shown direct toxicity of GT to assorted oral and gut bacteria during long-term incubation *in vitro* via membrane damage, prevention of biofilm formation, enzyme poisoning, etc.^32,33^. Members of the *Streptococcus* genus tend to be particularly susceptible to GT polyphenol toxicity *in vitro* as are some species of *Staphylococcus* though somewhat less so. The concentration range for GT effects on bacteria can vary from 10–1000 µg/ml^32,34–36^. This is well within the range of GT catechin concentration in undiluted tea (approximately 1 mg/ml at most). There is limited knowledge about selective antibiotic effects of GT consumed on bacteria *in vivo* and almost all studies were in gut. *In vivo* bacteria are typically present in biofilms and are therefore likely to be exposed to lower levels of GT-derived compounds. Studies focusing on a limited number of genera, in animals where GT extract or GT polyphenols are added to food at 0.2 to 0.6%, have shown decreases in gut *C*. *Perfringens* and other genera and increases in *Bifidobacterium* and *Lactobacilli*^37–39^. A study of humans consuming a tea polyphenol mix (largely of catechins) at the equivalent of 10 cups per day saw reduced *C*. *perfringens* and other *Clostridium* species while *Bifidobacterium* increased^40^. A much more recent study with ten subjects consuming 10 cups of GT per day saw an increase in *Bifidobacterium*^41^. A study that examined effects of high-level dosage of EGCG plus caffeine over a 12-week period in humans saw no changes in gut microbiota on the phylum level in 30 subjects compared to baseline but did not test for changes on lower classification levels of bacteria^42,43^. As evidence mounts that the oral cavity is a source of bacteria that play a role in systemic disease, it becomes important to determine if GT consumed at a reasonable level can alter the oral microbiome and thus oral and systemic health.

Soft tissue disease in the oral cavity is often related to tobacco exposure. We hypothesized that GT exposure would result in measurable changes in oral epithelial miRNA expression and oral microbiome in smokers. The detection of such changes is a first step in identifying mechanisms by which GT exposure can affect oral health. We enrolled a group of 16 subjects screened to ensure low baseline catechin and polyphenol levels and no tea exposure. Over a 4-week period the subjects consumed five cups of GT per day, with sampling for oral epithelial miRNA expression and oral microbial community structure analyses done at intervals.

## Results

### Tobacco users show marked changes in miRNA expression in oral epithelium

16 tobacco-using subjects were enrolled. One dropped out due to scheduling problems. Based on the food frequency questionnaires (FFQs), all showed low level of dietary intake of polyphenol at the start of the study (Table 1). RNA was isolated from the lateral border of the tongue. RNA from the 11 subjects with the highest yield was studied, (4 females, 7 males) 22 to 47 years of age with average age of 35. Control samples from nonsmokers (5 females, 4 males) 28 to 77 years of age, were also largely from the lateral border of the tongue, though two samples were from keratinized gingiva^44^. A comparison of miRNA expression between the two groups revealed many differences in the smoker group even in normal appearing tissue. Tobacco exposure is associated with marked differences in epithelial gene and miRNA expression^45,46^, and that was what was observed. Comparison to an earlier study on bronchial epithelium revealed that of 12 miRNAs detectable in both studies, 3 showed the same statistically validated differences in oral epithelium with tobacco smoke exposure, miR-126-3p, miR-133a-3p, and miR-193-3p^22^ (Table 2). That the miRNA changes were substantial in tobacco smoke exposed lateral border of the tongue was corroborated by the fact that nonsupervised clustering of the samples^47^ correctly sorted them into smoker and nonsmoker samples with 100% accuracy (Supplementary Fig. S1).

**Table 1:** Demographics and usual dietary intakes of 15 enrolled participants who completed the study.

Characteristics	Total Sample *N* = 15	Women (*n* = *6*)	Men (*n* = *9*)
Age (y)	31.3 ± 7.3	31.2 ± 6.4	31.4 ± 8.0
BMI (kg/m^2^)	25.5 ± 5.2	28.2 ± 8.1	24.0 ± 2.1
**Reported Activity levels (%)**			
Low active/sedentary	65	100	45.4
Active/very active	35	0	54.5
Multivitamin Supplement use (%) baseline-	47.1	33.3	54.5
***Daily Food Intakes***			
Energy	2568 (1789, 3332)	1789 (1353, 2234)	3186 (2101, 3643)
Dietary fiber, g/1000 kcal	8.6 (5.7, 9.2)	7.3 (5.3, 9.5)	8.8 (5.9, 9.2)
Fruit (svgs)	0.5 (0.2, 0.8)	0.2 (0.0, 0.7)	0.8 (0.2, 0.8)
Vegetables (svgs)	0.5 (0.2, 1.5)	0.2 (0.0, 1.2)	0.5 (0.2, 2.0)
Whole Grains (svgs)	0.5 (0.1, 1.5)	0.1 (0.2, 2.3)	0.7 (0.2, 1.4)
Low Fat Dairy (svgs)	0.2 (0.0, 0.8)	0.1(0.0, 0.6)	0.2 (0.1, 1.3)
Sweets (svgs)	1.0 (0.5, 1.3)	1.2 ((0.6, 2.2)	0.8 (0.3, 1.1)
Alcohol (% energy)	6.7 (2.7, 16.1)	3.3 (0.0, 22.0)	7.1 (4.4, 15.5)
HEI 2010 score	55.2 (50.5, 63.6)	56.8 (46.2, 64.4)	55.2 (50.4, 64.9)
Total Polyphenols (mg)	2.1 (0.6, 14.5)	1.3 (0.2, 5.8)	6.7 (0.8, 27.0)
(mg/1000 kcal)	1.1 (0.3, 6.9)	0.7 (0.2, 2.8)	2.7 (0.2, 7.4)

BMI: Body mass index; HEI 2010 Score: Healthy Eating Index 2010 score developed by the USDA^1^ to assess overall dietary quality with scores that range from 0 to 100, where 100 reflect optimal dietary quality^97^.

**Table 2:** Top miRNAs significantly up- and down-regulated in smokers vs never smokers FDR < 0.05.

Unique ID	Parametric p-value	FDR	Fold-change^1^
hsa-miR-185-5p	<1e-07	<1e-07	0.12
hsa-miR-31-5p	3.00E-06	0.000309	9.02
hsa-miR-133a-3p	8.00E-06	0.000549	51.27
hsa-miR-629-5p	3.71E-05	0.00171	3.19
hsa-miR-215-5p	4.15E-05	0.00171	2.32
hsa-miR-155-5p	5.01E-05	0.00172	0.26
hsa-miR-134-5p	6.27E-05	0.00185	5.66
hsa-miR-212-3p	0.0001077	0.00221	4.35
hsa-miR-34b-3p	0.0001095	0.00221	3.36
hsa-miR-34c-3p	0.0001128	0.00221	5.97
hsa-miR-19b-3p	0.0001178	0.00221	0.51
hsa-miR-181b-5p	0.0001344	0.0023	0.33
hsa-miR-502-5p	0.0001577	0.0025	8.52
hsa-miR-361-5p	0.0001717	0.00253	2.07
hsa-miR-132-3p	0.0004769	0.00655	2.29
hsa-miR-324-5p	0.0005343	0.00688	0.48
hsa-miR-197-3p	0.0005722	0.00693	2.6
hsa-miR-192-5p	0.000799	0.00866	2.08
hsa-miR-126-5p	0.000872	0.00898	7.7

^1^Fold change of 1 is no change.

### miRNA in lingual mucosa in tobacco users before and after GT exposure

Brush biopsy at the end of 4 weeks of tea consumption was used to harvest lingual epithelium from the lateral border of the tongue. An analysis of the expression level of 372 miRNAS revealed no average changes rose to the false discovery rate, FDR, of 0.1 and few even had uncorrected p values below 0.05. (Table 3, Supplementary Fig. S2).

**Table 3:** No miRNAs were significantly up- or down-regulated after 4 weeks of GT consumption. p < 0.05.

Unique ID	Parametric p-value	FDR	Fold-change^1^
hsa-miR-326	0.0245	0.981	0.54
hsa-miR-187-3p	0.0338	0.981	0.25

^1^Fold change of 1 is no change.

### Analysis of GT effects on coordinated miRNA expression in the oral epithelium

Rather than measuring mean changes in miRNA levels before and after GT exposure, a combined measure of differential gene expression and co-expression of miRNA pairs was done to identify co-expressed miRNAs that changed in level with GT exposure (see methods section). This analysis revealed 15 pairs of miRNAs that were co-expressed with GT exposure that also showed significance at FDR 0.01 using the Stouffer combined Z-score (Table 4). Of the 15 pairs, including 18 miRNAs, mir-181a-5p and mir-301a-3p formed hubs co-expressed with 7 and 3 other miRNAs, respectively (Supplementary Fig. S3). mRNA targets of these two groups of miRNAs were identified using Tarbase^48,49^. miRPath was then used with the group of mRNAs targeted by miR-181a-5p, miR-150-5p, miR-145-5p, miR-425, miR199b-5p, miR-328-3p, miR-766-3p, and miR-142-3p to identify a list of enriched mRNA-associated pathways, identified at p < 0.0001 of statistical significance. Any pathways that appeared at the same or higher significance when examining 40 groups of 8 random miRNAs were omitted. The Kyoto Encyclopedia of Genes and Genomes (KEGG) pathways, estrogen receptor signaling pathway hsa0915, central carbon metabolism in cancer, hsa05230, signaling pathways regulating pluripotency of stem cells, hsa04550 were highly associated with the miR-181a-5p hub miRNAs. The second group, of miR-301a-3p, miR-30e-3p, miR-182-5p, and miR-30e-5p showed no pathway that was not also identified using random lists of 4 miRNAs and miRPath.

**Table 4:** miRNAs pairs that are co-expressed and show evidence for differential expression by 4 weeks of GT consumption.

miRNA	miRNA2	P_DE (miRNA1)	P_DE (miRNA2)	CC	P_CC	Min_ttest_pval	Stoufer Zscore	pval_Zscore	FDR_Zscore
hsa-miR-328-3p	hsa-miR-181a-5p	3.61E-01	4.67E-02	7.74E-01	1.18E-05	4.67E-02	4.28	9.28E-06	7.21E-03
hsa-miR-199b-5p	hsa-miR-181a-5p	4.90E-01	4.67E-02	8.44E-01	3.94E-07	4.67E-02	4.73	1.14E-06	2.43E-03
hsa-miR-378a-3p	hsa-miR-375	3.07E-02	6.70E-02	6.93E-01	1.76E-04	3.07E-02	4.29	8.78-06	7.15E-03
hsa-miR-425-5p	hsa-miR-181a-5p	2.11E-01	4.67E-02	8.19E-01	1.55E-06	4.67E-02	4.82	7.16E-07	1.75E-03
hsa-miR-423-3p	hsa-miR-484	8.03E-02	2.53E-02	6.84E-01	2.25E-04	2.53E-02	4.24	1.14E-05	8.27E-03
hsa-miR-301a-3p	hsa-miR-30e-5p	4.29E-02	5.24E-01	7.94E-01	5.04E-06	4.29E-02	4.28	9.23E-06	7.21E-03
hsa-miR-301a-3p	hsa-miR-30e-3p	4.29E-02	3.61E-01	8 > 86E-01	2.08E-08	4.29E-02	5.32	5.07E-08	3.47E-04
hsa-miR-301a-3p	hsa-miR-182-5p	4.29E-02	2.78E-01	8.55E-01	1.95E-07	4.29E-02	5.08	1.85E-07	7.94E-04
hsa-miR-150-5p	hsa-miR-181a-5p	4.09E-01	4.67E-02	7 > 71E-01	1.35E-05	4.67E-02	4.21	1.30E-05	9.06E-03
hsa-miR-145-5p	hsa-miR-181a-5p	1.87E-01	4.67E-02	7.88E-01	6.76E-06	4.67E-02	4.60	2.11E-06	3.79E-03
hsa-miR-145-5p	hsa-miR-766-3p	1.87E-01	4.47E-02	7.22E-01	7.38E-05	4.47E-02	4.16	1.62E-05	9.92E-03
hsa-miR-181a-5p	hsa-miR-766-3p	4.67E-02	4.47E-02	7.15E-01	9.10E-05	4.47E-02	4.43	4.62E-06	4.60E-03
hsa-miR-181a-5p	hsa-miR-142-3p	4.67E-02	1.30E-01	7 > 70E-01	1.41E-05	4.67E-02	4.56	2.52E-06	3.87E-03
hsa-miR-766-3p	hsa-miR-142-3p	4.47E-02	1.30E-01	7.53E-01	2.65E-05	4.47E-02	4.45	4.23E-06	4.52E-03
hsa-miR-340-5p	hsa-miR-410-3p	1.27E-01	2.86E-02	−8.12E-01	2.27E-06	2.86E-02	4.99	3.08E-07	9.59E-04

p DE miRNA is p value of student t test of specific miRNA level at baseline, 0 weeks, compared to that after 4 weeks of GT consumption

CC is Spearman rank correlation of expression between the miRNA1 and miRNA2

Z Stouffer calculated by weighted sum of z-values assigned to p DE(mRNA1), p DE(mRNA2), and p CC.

### Changes in oral mucosal bacteria with GT consumption

Twelve subjects (6 females, 6 males, 26–48 years of age, average age 35) were subjected to oral swabbing of the lateral border of tongue and gingival mucosa. Ten subjects provided samples through the whole trial, while two had microbes collected only after the first week. On average for tongue samples there were 32,933 reads after chimeric removal per sample when we sequenced the 16S rRNA hypervariable region, with counts ranging from 14,927 to 48,270. For keratinized gingiva, there were 40,516 average, ranging from 16,254 to 80,508.

### Effect of GT on oral microbiome

**Figure 1:**
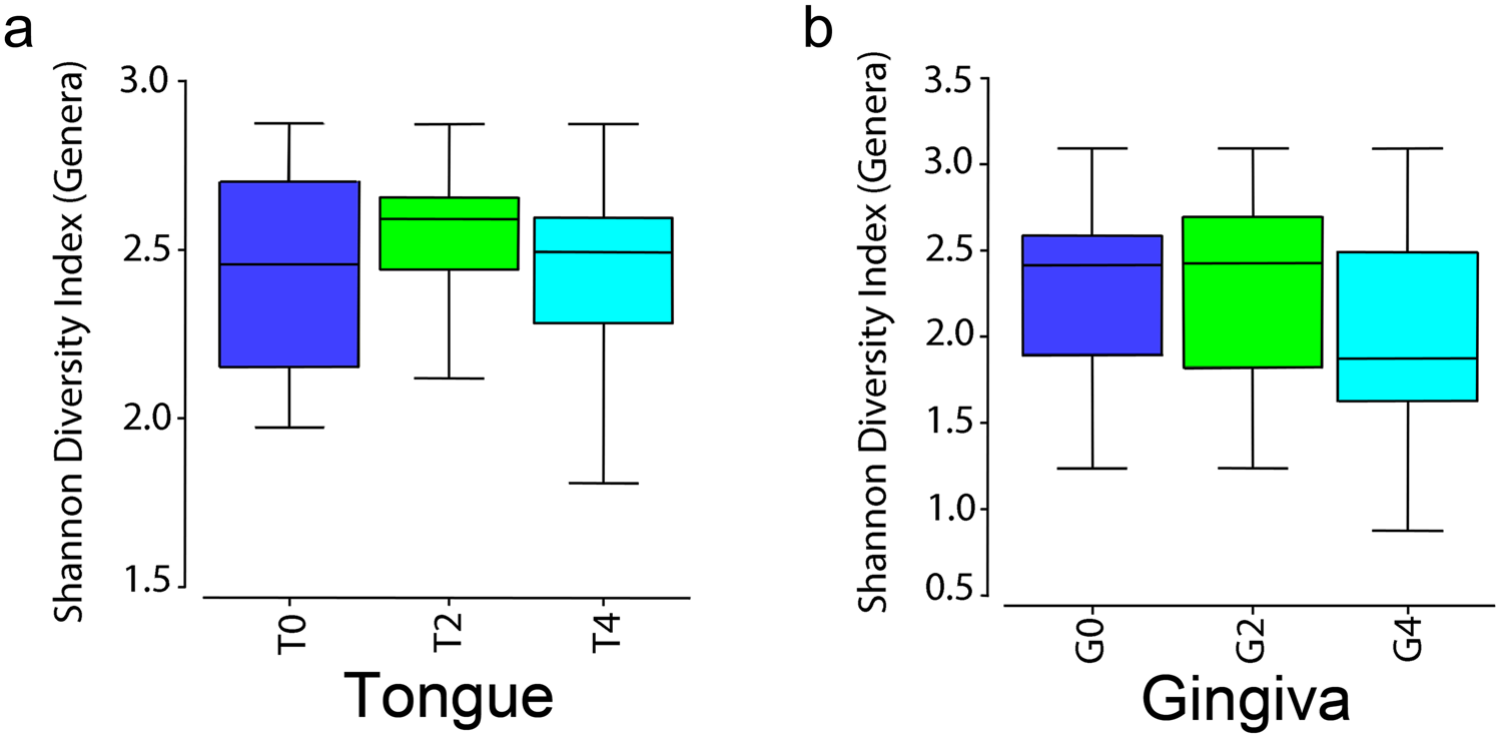
Shannon’s Diversity Index at the genus level for the different sites at baseline and after 2 and 4 weeks of 5× per day GT consumption did not differ. **(a)** Samples from lateral border of the tongue. **(b)** Samples from keratinized gingiva.

*In vitro* GT polyphenols can kill a large range of bacteria types, while some are resistant. Quantitation of the level of total bacteria 16s rRNA genes in each sample measured by qPCR before and after tea consumption provides an estimate of total bacteria content in samples. This was done for lingual samples and showed normalized levels of total bacteria 16s rDNA at 1.0 ± 0.34 week 0 and 0.80 ± 0.32 week 4, t < 0.55 by Student’s t-test. NGS sequencing of the same samples and QIIME based matching to known OTUs allowed quantitation of relative amounts of each OTU in each sample. Analysis of Alpha diversity by computing the Shannon Diversity Index revealed no difference in the richness or distribution of the bacterial genera on lingual or gingival surfaces in subjects at baseline versus after 2 and 4 weeks of GT exposure (Fig. 1). Together these findings suggest that GT as a diet component did not act as a broad-spectrum antibiotic.

Beta diversity comparisons allow us to examine differences overall in the identity of the bacteria on each surface before and after GT exposure. This tested if GT exposure, directly or indirectly, can causes change in the microenvironment that affects the selection process on these two oral mucosal surfaces thus changing the identities of taxa that are found. Non-metric multidimensional scaling (NMDS) was used to make these comparisons and revealed that overall the exposure to GT did not make large changes in the pattern of genera in the baseline versus post 4-weeks GT exposure at either site (Supplementary Fig. S4).

**Figure 2:**
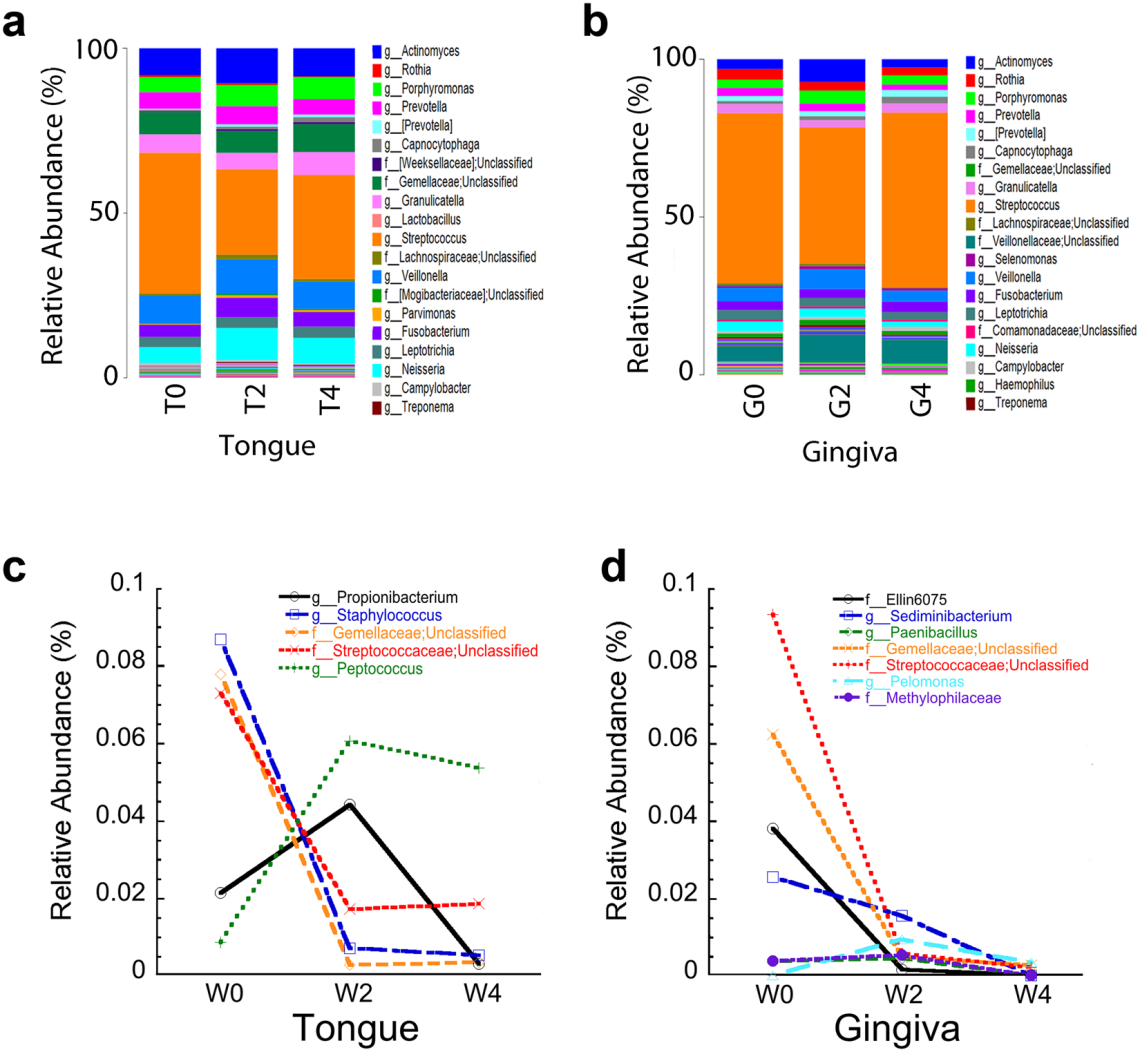
Taxonomic profiles averaged at baseline and at 2 and 4 weeks after GT exposure for **(a)** Tongue and **(b)** keratinized gingiva. We note *Streptococcus* is the dominant genus. **(c** and **d)** Levels of genera and subsets of families that were differentially abundant at 0 vs. 4 weeks of GT exposure are shown for tongue and keratinized gingiva, at FDR < 0.1 Note unclassified samples are subsets of families that were not assigned to a specific genus.

**Figure 3:**
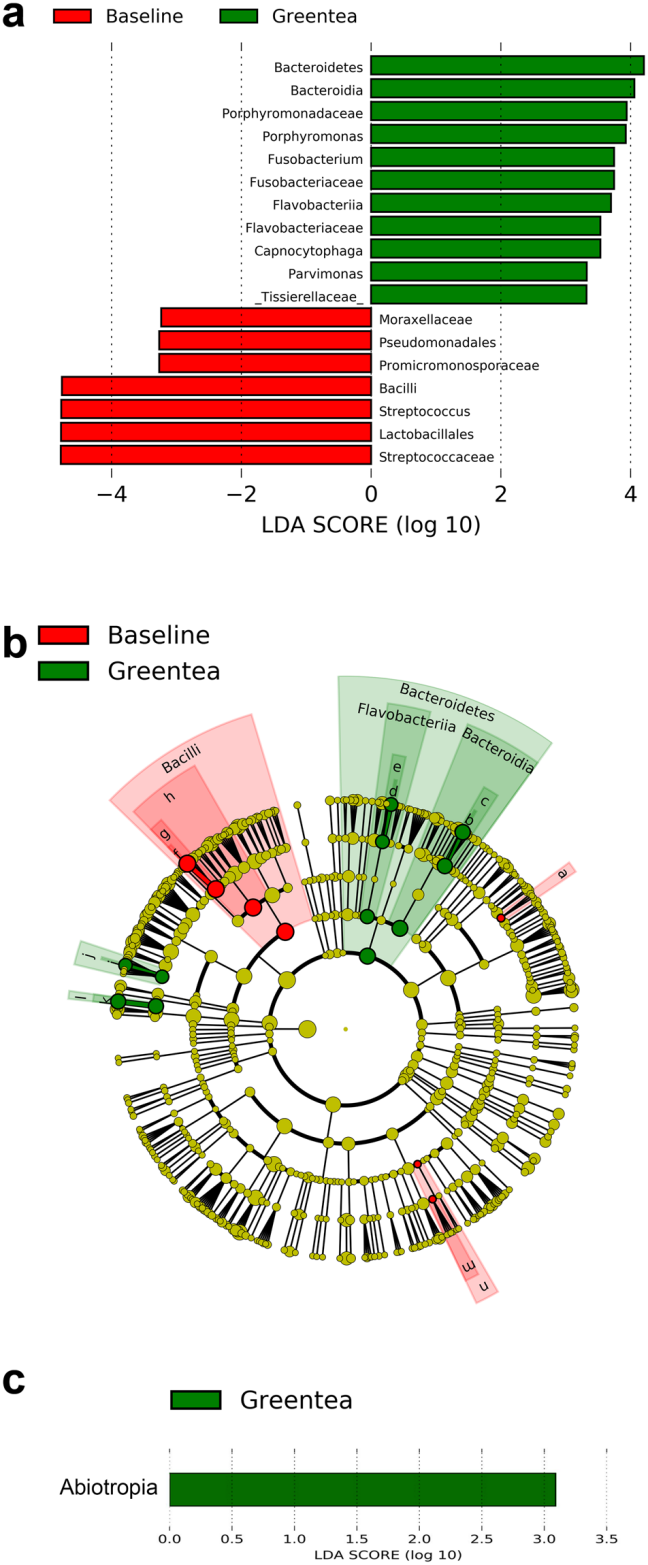
LDA Effect Size (LEfSe) algorithm was used on genus level OTU tables to determine taxa that best characterize the changes in the mucosal microbiome that occur at 0 versus 2 and 4 weeks of GT exposure. (**a**) Tongue showed decreases in *Streptococcus* and *Lactobacillus* among other changes. (**b**) The cladogram for the taxa that are different in tongue with green tea exposure shows their taxonomic relationships. (**c**) Keratinized gingiva showed less conclusive changes with only the genus *Abiotrophia* different at a high level of statistical significance.

An examination of only the major constituents of the microbiome before and during GT exposure on the two mucosal surfaces is shown in Fig. 2. The only discernable change is that in lingual tissue there is a decrease in *Streptococcus* during GT exposure (Fig. 3). More informative is a representation of the differences in all taxa present in Fig. 2C and D. We found significant differences in total abundance of a number of genera or subset of families when comparing values after 4 weeks of GT exposure to those from baseline using White’s nonparametric test and Storey’s FDR (p < 0.1) to correct for multiple testing^50,51^. Some of the taxa detected as differentially abundant were unclassified at the genus level. With the exception of one taxon, all showed consistent changes after 2 and 4 weeks of GT consumption (Fig. 2).

To determine the taxa changes most likely biologically relevant to GT exposure, we used linear discriminant analysis of effect size (LEfSe) to determine taxa that were differentially abundant before and after GT exposure comparing the baseline levels versus those at weeks 2 and 4 of GT exposure, which were combined into one group^52^. An examination of tongue bacteria showed changes in several bacteria groups, including a decrease in the high frequency genus *Streptococcus* on the lateral border of the tongue, while several taxon units at low levels at that site were shown to increase (Fig. 3). This method to highlight biologically relevant markers only showed one operational taxon unit to change with high statistical significance at the keratinized gingival site with GT consumption.

## Discussion

A major goal of this study was to define oral mucosal miRNA expression changes induced by GT exposure, which would shed light on the mode of action of GT on oral mucosal health. miRNA expression changes due to tobacco exposure were measured in oral epithelium. As a positive control miRNA expression in epithelium of the lateral border of the tongue in subjects who had smoked cigarettes over at least 2 years was compared to that of a separate group of never smokers (see Table 2). A large number of differences that were distinct enough to separate the samples based on smoking status with 100% accuracy using nonsupervised clustering were observed (Supplementary Fig. 1)^53^. Some of the changes were similar to those seen in earlier studies on other epithelium, such as that of the bronchi, including changes in miR-126-3p, miR-133a-3p, and miR-193-3p^22^. That not more were shared in both studies may be due to differences in the type of mucosa, squamous epithelium versus pseudostratified columnar epithelium or the method of miRNA measurement. This nonsmoker versus smoker comparison, a cross sectional study, showed differences in 19 miRNAs at FDR < 0.01% with several miRNAs showing 5 × differences. A similar statistical analysis revealed no consistent changes at FDR < 0.1 when the same cigarette smokers consumed 5 cups of GT per day over a 4-week period (see Table 3, Supplementary Fig. 3)^54^. The conclusion is that either GT had little effect on the oral epithelium RNA or that many of the changes were too variable across the group to show consistent differences.

Working on the assumption that some subjects may have oral epithelium that respond more robustly to GT than others, we hypothesized that some miRNAs may be co-regulated and show similar changes in level with GT exposure. Thus, subjects with low or intermediate GT response would not obscure the high level changes seen in the best responders if there was also a focus on maintenance of co-expression. Indeed, 2 hubs with co-regulated miRNAs were identified that show some level of differential expression with GT exposure. Interestingly, one of these hub miRNAs, miR-181a-5p, and the 7 co-expressed miRNAs, target mRNAs associated with the estrogen receptors signaling pathway. GT catechin have been shown to both inhibit or stimulate in a concentration dependent manner, different components of the estrogen receptor signaling pathway including ER alpha itself^55^ Estrogen receptor and estrogen levels are thought to be important in head and neck SCC outcomes^56^. The epidermal growth factor receptor is activated during carcinogensis in many head and neck cancers and is thought to be a key driver of that state^57^. Two additional pathways identified, which may be targeted by the hsa-miR-181a-5p hub, are central carbon metabolism in cancer and signaling pathways regulating pluripotency of stem cells, and are also potentially directly or indirectly regulated by green tea consumption, and may play roles in its OSCC inhibition^58,59^, but much more work is needed to verify these GT-related miRNA changes and GT effects on the 3 pathways.

Changes in oral mucosal bacteria with GT exposure were clear in our study. Most of the changes, before and after tea exposure, were different at the two mucosal sites tested. Lateral border of the tongue and keratinized gingival epithelium, though both squamous epithelium, have different histological features and indeed showed different adherent microbiomes^60^. Because biofilms on different mucosal surfaces may depend on different anchor bacteria, it is hard to predict how GT will affect different biofilms. It was not clear if changes in certain genera were directly due to cytotoxicity, changes in bacteria adherence, or effects on other bacteria in the biofilm, etc. Importantly, the time course in Fig. 3 showed similar changes in levels of OTUs at both 2 weeks and 4 weeks of GT exposure, reinforcing the identification of these OTUs as being differentially abundant after GT exposure. While they did not mimic the changes seen in earlier studies on gut, that was not expected, due to all the differences in the gut versus oral environment^37–41^. GT did not seem to work as a broad-spectrum antibiotic as a reduction in total bacteria and/or a decrease in bacterial diversity was not seen (Fig. 1).

LEfSe analysis, which in part puts the focus on changes in closely related bacteria types, highlighted decreases in *Streptococcus* after GT exposure^52^. *Streptococcus* is a major constituent of lingual and gingival mucosa biofilm so if this change is a common response to GT exposure it has the potential to have large effects on oral mucosa. *Staphylococcus*, typically at low level in the mouth, was also observed to decrease after GT exposure. Both of these genera have been shown to be elevated in the mouths of tobacco users and oral squamous cell carcinoma patients, so these genera are associated with habits that lead to OSCC and possibly OSCC itself^61,62^. Tobacco smoke is thought to change oral bacteria by altering oral pH, O_2_ levels, bacteria adherence properties, host immunity, or oral nutrients^62–66^. Because *Streptococcus* and *Staphylococcus* are associated with tobacco use, one might propose reductions in these bacteria by GT could help inhibit OSCC formation. We note over 13 different *Streptococci* species were identified in the samples by metagenomic analysis, making it difficult to pinpoint the species affected by GT. Several genera were shown to increase with GT exposure and LEfSe highlighted some of these. *Porphymonas gingivalis* and *Fusobacterium nucleatum* are species of interest in OSCC as they are associated with other cancers and our in the oral cavity^67,68^. *Porphyromonas*, seen to increase after GT consumption, likely refers to two species *KLE1280* and *oral taxon 279* and not *Porphyromonas gingivalis*, which is known to be inhibited by GT extract and was present at 100× -fold lower levels when detectable in the tongue mucosal samples^35^. The most abundant *Fusobacteria* was *Fusobacterium periodontium*, a species not associated with carcinogenesis. Several other *Fusobacterium* species, including *Fusobacterium nucleatum*, which has been associated with colorectal cancer and is enriched on OSCC mucosa^68–70^ were present at much lower levels on the lateral border of the tongue. While GT is directly toxic to *Fusobacterium nucleatum in vitro*, causing killing and changing adherence^71^, we do not have measurements of the levels of this particular species after GT exposure in our studies. LEfSe analysis of gingival bacteria was less clear and only one genus showed differential abundance at high probability after 2 and 4 weeks of GT exposure at that site.

As postulated some years ago, GT and other polyphenols may work largely indirectly in the human body^72^. First, as shown here, they can change oral bacteria, which may change oral and systemic health. Second, it appears GT chemicals, such as the catechins, are much more efficiently absorbed by gut mucosal epithelium, for example, and then transferred to the blood, after metabolism by colonic microbes^73,74^. These metabolites have higher bioavailability than the parent molecule^14,15,75^. Metabolism of GT catechins also occurs in the mouth but the process is not well described^12,76^. One explanation for variable oral epithelial response to GT may be differences in oral bacteria or cells enzymes that metabolize GT to forms that can be absorbed. Our human results stand in contrast to those from Zhou *et al*. who saw consistent and significant changes in miRNA expression in the lung adenomas of mice given GT extract in their water over 1 week^18^. We speculate that mice, often genetically identical, housed together on the same diet, would have similar oral and gut bacteria, and would thus respond consistently to GT extract exposure in regard to RNA expression or, in turn, inhibition of epithelial tumor formation.

Care was taken to make sure polyphenol intake of the subjects was unchanged throughout the study, except for the consumption of the GT. An examination of the dietary information shows overall the group consumed minimal levels of polyphenol due to limited fruit and vegetable intake (Table 1). Urine EGCG examination showed variable baseline but increases in urine EGCG in all subjects except one (see Supplementary Table 1). Surprisingly, one subject who consumed tea immediately prior to sample acquisition showed exceptionally high aglycone EGCG in urine at two different times, suggesting very rapid excretion of much of this catechin right after consumption^14,77^. While the subjects involved in this study did not have an illness, thus minimizing some potential aspects of the placebo effect, it is possible the effects seen were due to the thermal effects of drinking warm water or the reduction of some other component(s) of their diet. Most importantly, very recent clinical work on another proposed neutraceutical, curcumin, has revealed gender specific effects on gene expression in blood monocytes^78^. Given that in one study that women, and not men, showed a negative correlation between GT consumption and OSCC it will be important to determine if GT has similar gender-specific miRNA expression effects in a study with sufficient numbers of subjects^7^.

## Methods

### Subjects

16 smokers (5–15 cigarettes per day during the preceding two years) were recruited. Subjects reported no usage of antibiotics or other medications in the two weeks preceding the study and through the course of the study. At entry all recruits completed a food frequency questionnaire (FFQ) used to estimate usual intakes of polyphenol-rich foods and to determine little or no tea intake in the month before the study (Graphical Vioscreen FFQ, Viocare, Princeton, NJ, USA)^79^. Nutrient composition analyses of FFQ using Nutrition Data Systems for Research, version 45, (University of Minnesota Nutrition Coordinating Center, Minneapolis, MN, USA) was performed within Viocare software. Additional data on polyphenol content in foods were obtained from the Phenol-Explorer database (www.phenol-explorer.eu)

The subjects were instructed to maintain their initial diet over the study, avoiding changes in fruits, vegetables, and other beverages enriched in polyphenols. After entry into the study, a dietary screener, which contained a checklist of these polyphenol-rich foods and beverages (i.e, wine, tea, coffee, berries, citrus fruits, beans, onions nuts, leafy vegetables, beer, fruits) was administered to all subjects weekly. All subjects provided RNA, the 11 with the highest yield were studied. Twelve subjects supplied oral microbiome samples at various time points.

All subjects in all groups provided oral informed consent for the telephone interview to determine eligibility and then written informed consent to participate in accordance with guidelines of the Office for the Protection of Research Subjects of the University of Illinois at Chicago, the local Institutional Review Board that formally approved of this research. Control nonsmokers used for RNA studies were described in an earlier study (34).

### Tea dosage

All subjects were instructed on tea preparation and given 35 tea bags of Bigelow Organic Green Tea, each bag steeped in 1 cup water 1 minute at 80 °C. Subjects were encouraged to allow the tea to cool several minutes prior to drinking. Subjects returned every 7 days and were given tea for the next week and returned all used tea bags. The tea used was selected because it had the higher EGCG level of two brands tested that were available in single lots^80^.

### Measurement of aglycon EGCG in urine

All urine samples were collected at the subjects’ first visit and then at weekly intervals between 10:00 AM and 4:00 PM typically the same time for each subject. Samples were aliquoted and stored at −80 °C with 100 µM ascorbic acid. Liquid chromatography followed by tandem mass spectroscopy was used to separate and analyze levels of EGCG after enzymatic cleavage from glucuronide and sulfate forms^80^.

### RNA analysis

Brush biopsy was used to harvest cells from the lateral border of the tongue of each subject and samples were stored in Trizol at −80 degrees prior to purification of small RNAS using RNeasy chromatography as described earlier. 100 ng RNA was reverse transcribed in 20 ul reactions using the miRCURY LNA Universal RT microRNA PCR, Polyadenylation and cDNA synthesis kit (Exiqon, Woburn, MA, USA) and was assayed once by RT-qPCR on the microRNA Ready-to-Use PCR, Human panel I (Exiqon), which includes 372 miRNA primer sets. Both version V3.M and V4.M panels were used. To compensate for individual miRNA assays that were altered by the manufacturer between versions, batch effect correction was done for all assays using Combat after preliminary normalization of tests within each group^81^. Negative controls, excluding template from the reverse transcription reaction, were tested and profiled like the samples with individual primer pairs. The amplification was performed in an Applied Biosystems Viia 7 RT-qPCR System (Life Technologies, Carlsbad, CA, USA) in 384 well plates. The amplification curves were analyzed for Ct values using the built-in software, with a single baseline and threshold set manually for each plate (Exiqon).

### Microbiota

#### Mucosal sample collection and analysis

Swab samples taken for microbiome analysis were frozen immediately after collection. Genomic DNA was extracted from swabs using the MasterPure Gram Positive DNA Purification Kit (Epicentre, Madison, WI, USA) according to manufacturer’s instructions.

#### Quantitation of microbial abundance

Quantitative PCR analysis was performed to determine the relative abundance of microbial 16 S rRNA genes in swab sample extracts. Amplification reactions were performed as described previously^82^. PCR was performed using a StepOne Plus Real Time PCR System (Life Technologies), and all samples were analyzed using technical duplicates.

#### Characterization of microbial community structure

Microbial community structure was characterized using high-throughput sequencing of PCR amplicons generated from the V1–V3 variable regions of bacterial 16S ribosomal RNA (rRNA) genes. Briefly, the widely used primer sets 27F/534R, targeting the V1–V3 variable region of the 16S rRNA gene of bacteria, was used for amplification as done earlier with slight modifications^83^. A two-stage PCR or “targeted amplicon sequencing (TAS)” approach was performed to generate amplicon libraries^84,85^. In the first of the two-stage amplification procedure, the templates were amplified (28 cycles) using primers containing 27F and 534R (Bacteria), and 5′ linkers CS1 and CS2 linkers, as described previously^86^ PCRs were performed in 10 µl reaction volumes using the KAPA HiFi HotStart PCR Kit, and the PCR conditions were as follows: 5 min initial denaturation at 95 °C, followed by 28 cycles of: 95 °C for 30″, 50 °C for 30″, 72 °C for 60″. Subsequently, a second PCR reaction was established, with one µl of amplification product from the first stage used as input to the second reaction. The primers for the second stage amplifications were the AccessArray barcoding system primers (Fluidigm, South San Francisco, CA, USA), containing Illumina sequencing adapters, sample-specific barcodes, and CS1 and CS2 linkers. PCR conditions for the second reaction were as follows: 5 min initial denaturation at 95 °C, followed by 8 cycles of: 95 °C for 30″ 60 °C for 30″, 72 °C for 60″. Samples were pooled in equimolar ratio and quantified using a Qubit 2.0 fluorometer. Sequencing was performed on an Illumina MiSeq sequencer using standard V3 chemistry with paired-end, 300 base reads. Fluidigm sequencing primers, targeting the CS1 and CS2 linker regions, were used to initiate sequencing. Demultiplexing of reads was performed on instrument. Library preparation was performed at the DNA Services Facility at the University of Illinois at Chicago, and sequencing was performed at the W.M. Keck Center for Comparative and Functional Genomics at the University of Illinois at Urbana-Champaign (UIUC).

### Bioinformatics analysis

#### Microbiome

Raw paired-end FASTQ files were merged using the Paired-End reAd merger (PEAR) algorithm^87^. Subsequently, merged data were quality trimmed (Q20) and sequences shorter than 450 bases were removed. The remaining sequences were exported as FASTA and processed through the software package QIIME (v1.8.0)^88^. Sequences were screened for chimeras using the USEARCH61 algorithm^89^ and putative chimeric sequences were removed from the data set. Chimera-free samples were then pooled, and clustered into operational taxonomic units (OTU) at 97% similarity using the usearch61 *de*^47^ generated at taxonomic levels from phylum to species.

### Statistical analysis

#### Epithelium RNA

Differential Expression Analysis: A two sample t-test was done to identify likely differentially expressed miRNAs with a parametric p-value derived and False Discovery Rate, FDR, to correct for multiple testing within the framework of BRB-Array Tools. The same was used to generate heat maps to display their expression after hierarchical clustering^53^. Only miRNAs detectable in over 60% of samples were examined.

Co-expression Analysis: miRNA expression levels of 11 subjects at baseline and 4 weeks were analyzed. miRNAs with more than 12 missing values out of the 22 samples were removed, and among the remaining miRNAs, missing values were replaced with the overall average of available expression values for the miRNA. For each miRNA differential expression between 4 wk and 0 wk, samples were tested using a two-sided paired t-test, and resulting p-value denoted by p_DE_. For each pair of miRNAs the Spearman correlation coefficient across all 22 samples was computed and the resulting p-value denoted by p_CC_. To combine evidence of co-expression of a miRNA pair with evidence of their differential expression, the Stouffer’s weighted z-score was used^90,91^.

1 $$\,Z(miR1,miR2)=\,\frac{{w}_{DE}{Z}_{DE}(miR1)+{w}_{DE}{Z}_{DE}(miR2)+{w}_{CC}{Z}_{CC}(miR1,\,miR2)}{\sqrt{{w}_{DE}^{2}+{w}_{DE}^{2}+{w}_{CC}^{2}}}$$

where Z_DE_(miR) denotes the Z-score of differential expression of miR obtained from its p_DE_ by applying the inverse of standard normal cumulative distribution function to 1- p_DE_ and Z_CC_(miR1, miR2) denotes the Z-score of co-expression of miR1 and miR2, similarly obtained from p_CC_. The weights used to combine evidence were w_CC_ = 1 and w_DE_ = 0.5. The Stouffer’s z-score was converted to a p-value using the standard normal distribution and finally corrected for multiple hypothesis testing using FDR. miRNA pairs at FDR < 0.01 where at least one miRNA of the pair has p_DE_ <0.05 were determined.

### Prediction of miRNA targets and their functional analysis

Potential miRNA targets were identified using DIANA‐miRPath v3.0 (http://www.microrna.gr/miRPathv3). Validated mRNA targets were selected, using Tarbase, which uses only experimentally verified targets^48,49,92^. For functional annotation of potential targets, we used Kyoto Encyclopedia of Genes and Genomes (KEGG) pathways term enrichment analysis, using the computational tool miRPath, to identify a list of pathways showing statistically higher levels of representation than that expected by chance. The Estrogen Receptor Signaling Pathway, hsa04915, was ranked highest using the experimentally verified Tarbase mRNA target sets and an adaptation of miRPath to help insure accuracy^93,94^. A similar analysis of 40 random sets of 8 miRNAs, was done and none were associated with such high probability with this pathway helping to show its validity^94^. Two other pathways, hsa05230 and hsa04550, were also identified though at lower probability. For each, one of forty randomized miRNA sets showed a similar probability of association with that pathway.

### Microbiome

BIOMs were used: (a) for calculation of alpha diversity indices (*e*.*g*. taxon richness), (b) for visualization of data at multiple taxonomic levels with non-metric multidimensional scaling (NMDS), (c) for analysis of similarity (ANOSIM) tests to determine if microbial communities were significantly different between groups, and (d) to identify taxa which were significantly differentially abundant between *a priori* defined groups. An unpaired Student’s *t* test was used to determine if microbial alpha diversity (*i*.*e*., Shannon index) was significantly different (*P* < 0.05) between smokers and non-smokers. Differences in microbiota taxonomic abundance between the groups were tested using White’s nonparametric test. False discovery rate–corrected *P* values were estimated in the method of Storey for all taxa comparisons^50^. Significance was set at *P* < 0.05 for White’s nonparametric test with a false discovery rate of <0.10 allowed^51^. All statistical analyses were performed using Primer 7^95^ with the exception of the identification of differentially abundant taxa, which was done using the software package STAMP^96^.

### Data availability

The sequencing data from this study is deposited as SRP136940 in the Sequence Read Archive at the NCBI.

## Electronic supplementary material

Supplementary Information
