# Supplementary material for: Effects of green tea on miRNA and microbiome of oral epithelium
Source: Sci Rep. 2018 Apr 12;8:5873. doi: 10.1038/s41598-018-22994-3 (PMC5897334; doi:10.1038/s41598-018-22994-3)
Supplement: Supplementary file 1 — Supplementary Information [file 41598_2018_22994_MOESM1_ESM.pdf]

Supplemental Information

Effects of green tea on miRNA and microbiome of oral epithelium

Guy R. Adami*, Christy C. Tangney, Jessica L. Tang, Yalu Zhou, Saba Ghaffari, Ankur Naqib, Saurabh Sinha, Stefan J. Green, Joel L. Schwartz

*Corresponding author: Guy R. Adami, E-mail: gadami@uic.edu

Supplemental Fig. S1 Hierarchical clustering of samples from current smokers and never smokers based on miRNA levels. Hierarchical clustering visualization of both samples and miRNAs was done after median centering and normalization of miRNA values and using centered correlation and average linkage. Control samples from never smokers (5 females, 4 males) 28 to 77 years of age, were largely from the lateral border of the tongue like smoker samples, though two samples were from keratinized gingiva. Smoker subjects are described in the text. This comparison for miRNA expression between the two groups revealed many differences in the smokers even in normal appearing tissue.

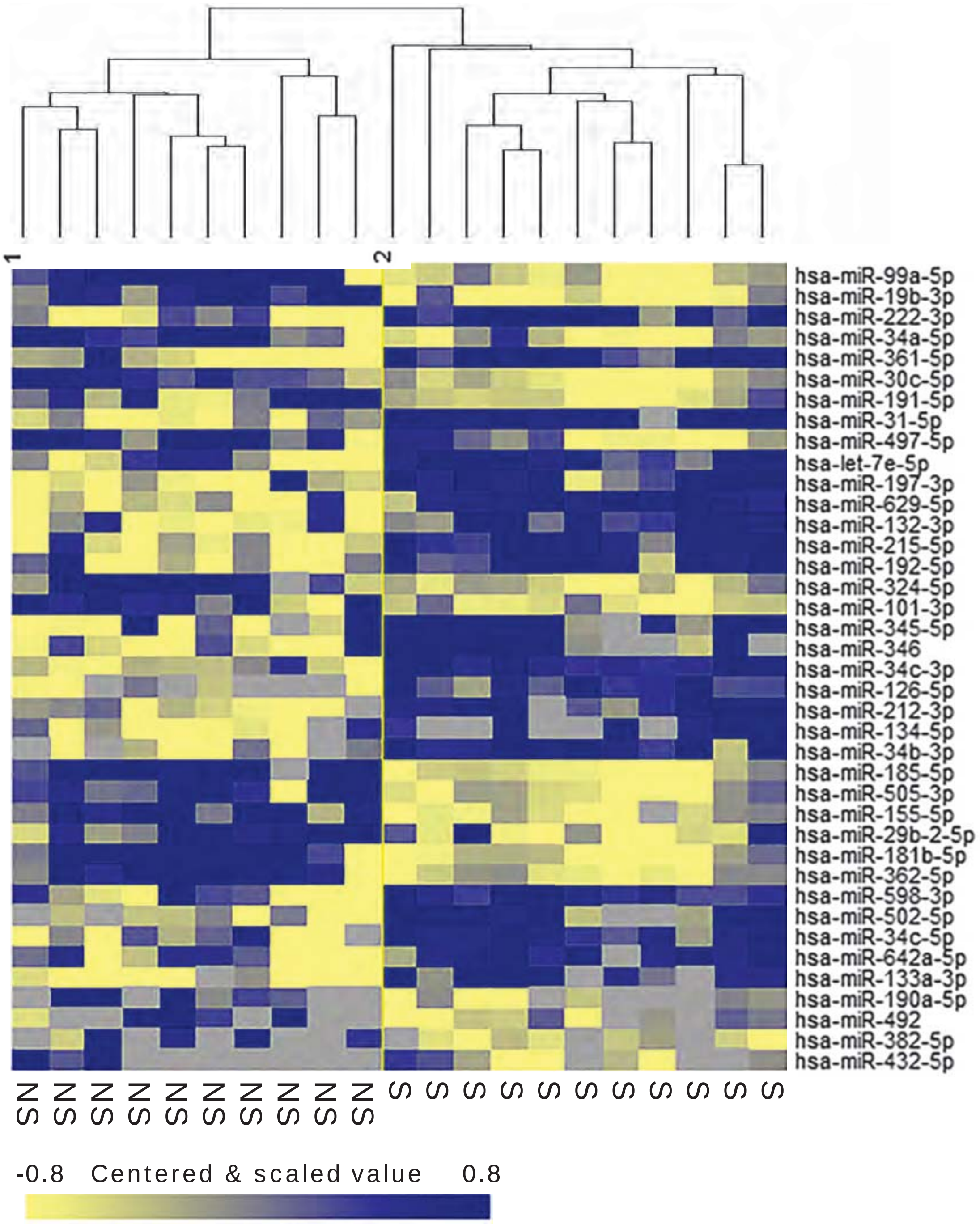

Data range before thresholding -3.6 to 3.6
Missing values are in color gray

Supplemental Fig. S2 Hierarchical clustering of samples from current smokers prior to green tea consumption and after 4 weeks of green tea consumption show poor correlation with treatment. Hierarchical clustering visualization of both samples and miRNAs was done after median centering and normalization of miRNA values and using centered correlation and average linkage.

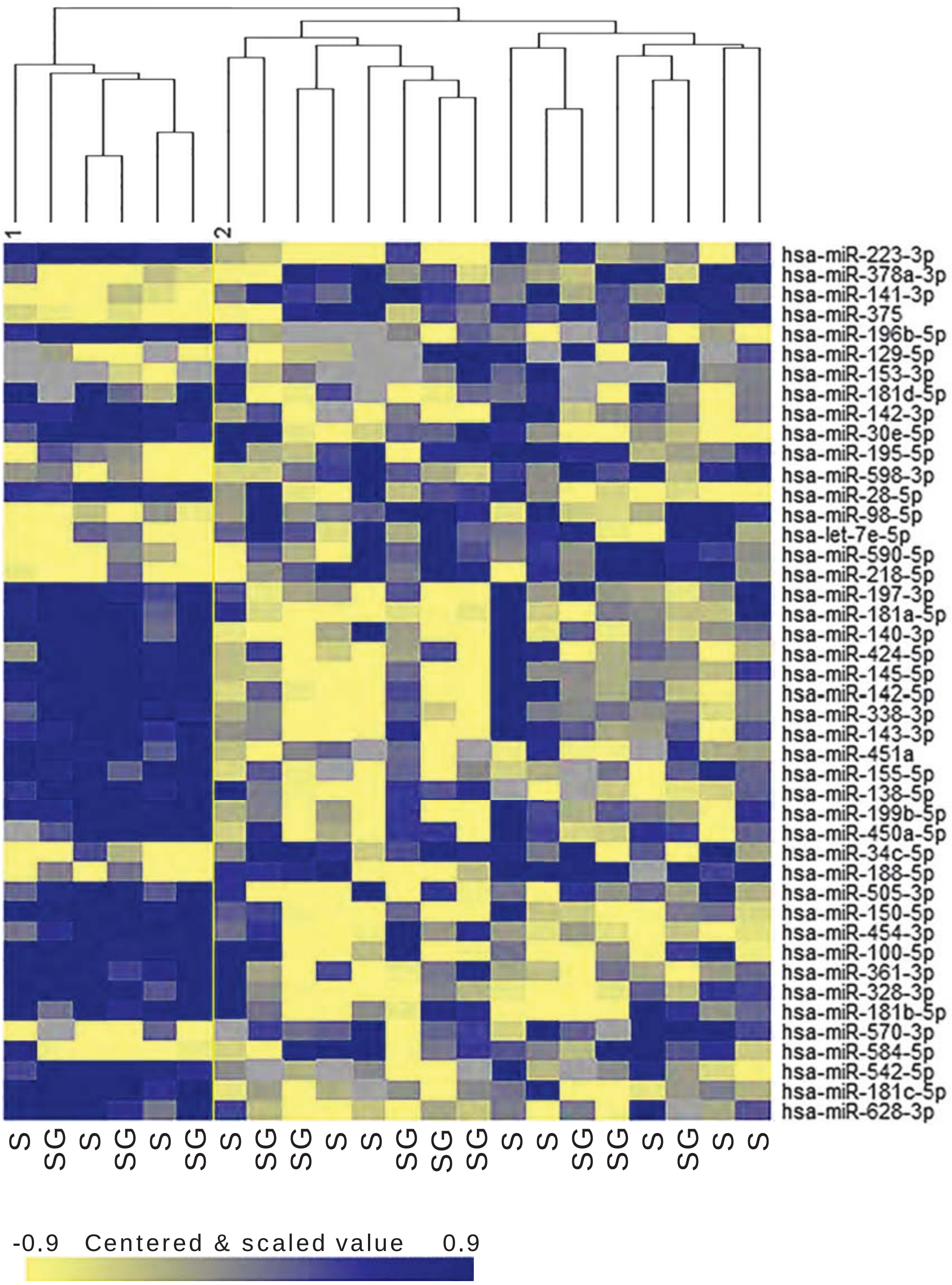

Data range before thresholding -2.8 to 2.6
Missing values are in color gray

Supplemental Fig. S3 Co-expression analysis revealed miRNA pairs that are coordinately expressed with one or both showing evidence for differential expression with green tea consumption. Each edge represents a co-occurrence relationship. All correlations in expression are postive with the exception of that between hsa-miR-340-5p and hsa-miR-410-3p.

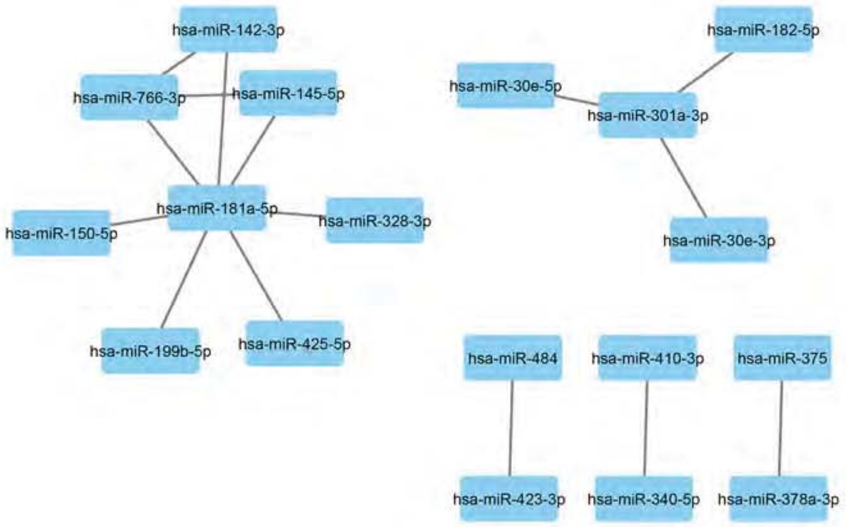

Supplementary Fig. S4 Beta diversity comparisons were done for the microbes at mucosal sites before and after GT exposure. Principal Component Analysis (PCA) using non-metric multidimensional scaling (NMDS) with Euclidean distances was not able to discriminate the datasets at either mucosal site.(a) Lateral border of the tongue, 10 samples at baseline and 10 samples after 4 weeks GT exposure. (b) Keratinized gingival, 10 samples at baseline and 10 samples after 4 weeks of GT exposure.

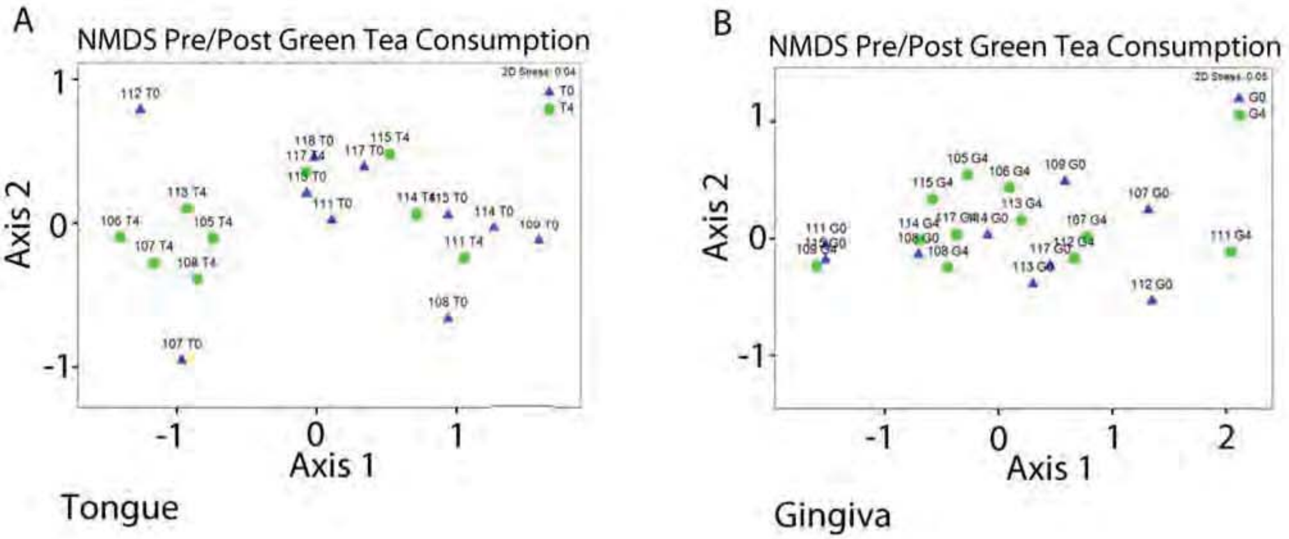

Supplementary Table S1 Subjects showed an increase in urine EGCG metabolites consistent with tea consumption

Sample	Week 0 (ng/mL)	Week 4 (ng/mL)
100	1.68 ± 0.07	11.1 ± 0.52
101	(<LOQ) 0.18 ± 0.02	5.67 ± 0.1
102	(< LOQ) 0.17 ± 0.03	2.34 ± 0.06
104	(<LOD) 0 ± 0	4.77 ± 0.13
105	3.39 ± 0.18	5.83 ± 0.1
106	2.86 ± 0.33	20.5 ± 0.57
107	4.58 ± 1.11	77896.25 ± 717.68
108	0.7 ± 0.17	25.54 ± 0.41
111	12.15 ± 0.19	21.34 ± 0.98
112	5.66 ± 0.37	8.22 ± 0.62
113	0.84 ± 0.03	4.76 ± 0.27
114	(<LOD) 0 ± 0	4.35 ± 0.21
115	4.04 ± 0.53	14.16 ± 0.91
117	4.12 ± 0.19	63.78 ± 1.11
